# Characteristics of Cerebral Infarction Due to Anterior Cerebral Artery Dissection: A Review of 16 Cases

**DOI:** 10.7759/cureus.80688

**Published:** 2025-03-16

**Authors:** Kenshi Sano, Atsushi Kuge, Tetsu Yamaki, Kosuke Sasaki, Rei Kondo, Yukihiko Sonoda

**Affiliations:** 1 Department of Neurosurgery, Yamagata City Hospital Saiseikan, Yamagata, JPN; 2 Department of Emergency Medicine, Yamagata City Hospital Saiseikan, Yamagata, JPN; 3 Department of Neurosurgery, Yamagata University, Faculty of Medicine, Yamagata, JPN

**Keywords:** anterior cerebral artery dissection, antihypertension therapy, cerebral angiography, cerebral infarction, vessel wall image

## Abstract

Objective: Intracranial artery dissection is a recognized cause of cerebral infarction and subarachnoid hemorrhage. In Japan, anterior cerebral artery dissection (ACD) represents one of the causes leading to ischemia. This study compares our clinical experience with existing literature to characterize the distinctive features of ischemia-onset anterior cerebral artery dissection (infarction-anterior cerebral artery dissection (iACD)).

Materials and methods: We conducted a retrospective analysis of 16 iACD cases identified among 2,776 cerebral infarction patients hospitalized between 2010 and 2019. Comprehensive imaging evaluations were performed at onset and systematically followed at two weeks, four weeks, one month, three months, six months, and 12 months post-onset.

Results: The patient cohort had a mean age of 59.1 ± 11.2 years, with six patients (37.5%) being female. Clinical presentation included aphasia in four patients (25.0%), while headache, typically considered characteristic of dissection, was reported in only one patient. Magnetic resonance imaging demonstrated intramural hematoma in 10 patients (62.5%), all confirmed during the second week following onset. Therapeutic management consisted of blood pressure control in all cases, with adjunctive antithrombotic therapy using cilostazol administered to five patients (31.3%). No patients developed progression to dissecting aneurysms or subarachnoid hemorrhage. While radiological deterioration occurred in the form of worsening stenosis (11 patients, 68.8%) and enlargement of cerebral infarcts (15 patients, 93.8%), and clinical deterioration manifested as worsening neurological symptoms in four patients (25.0%), none of these adverse events correlated with long-term prognosis.

Conclusion: Our findings diverge from previous reports in two significant aspects: iACD affects elderly populations more commonly than previously recognized, and the classic presenting symptom of headache is notably infrequent. Clinicians should maintain a high index of suspicion for ACD when evaluating cerebral infarction in the anterior cerebral artery territory, even in the absence of typical findings such as headache or age.

## Introduction

Anterior cerebral artery dissection (ACD) is one of the causes of cerebral infarction in the anterior cerebral artery region. [1]. A 2016 meta-analysis reported the following characteristics of ischemia-onset anterior cerebral artery dissection (infarction-anterior cerebral artery dissection (iACD)): (1) young onset (mean age 51 years), (2) many Asian patients (89 of 91 cases reported), (3) relatively high male prevalence, (4) 53% of patients have headaches, (5) characteristic neuroradiological findings were not always present, (6) double lumen, pearl and string, and intra-mural hematoma (IMH) in 32%, 60%, and 20%, respectively, (7) 90% of patients were treated conservatively, and (8) 77% of patients had a good prognosis [2]. However, the pathophysiology of cerebral infarction has been changing with the aging of the population in recent years [3]. In the present study, we experienced 16 cases of iACD over a 10-year period. We compare our experience with previous reports and discuss the clinical features of iACD.

## Materials and methods

A total of 2,776 consecutive patients admitted to the stroke center of Yamagata City Hospital Saiseikan, Yamagata, Japan, for ischemic stroke between January 2012 and December 2021 were included, and clinical information was collected and examined. Clinical information collected included age, gender, neurological symptoms, comorbidities, drinking history, smoking history, diagnosis based on the National Institute of Neurological Disorders and Stroke (NINDS) classification, lesions classified according to vascular territory, and modified Rankin Scale (mRS). The following imaging findings were examined in patients with lesions confined to the unilateral anterior cerebral artery region: magnetic resonance imaging (MRI) and digital subtraction angiography (DSA) at admission, two weeks, four weeks, three months, six months, and 12 months later. A follow-up MRI was performed at two weeks, four weeks, three months, six months, and 12 months later, and DSA was conducted twice after normalization. If normalization was confirmed twice, MRI follow-ups were conducted annually. All imaging findings were evaluated by two or more neurosurgeons or neuroradiologists using the picture archiving and communication system (PACS) system. The diagnostic criteria of arterial dissection proposed by Takagi et al. from a multicenter study in Japan were used [4]. The segmentation of the anterior cerebral artery was based on Salamon's classification, which is widely known [5]. Intramural hematoma (IMH) was defined as a high signal around the flow void on a non-contrast motion-sensitized driven-equilibrium sequence (MSDE). All patients were treated conservatively. Those with multiple risks of atherosclerosis were considered for treatment with antiplatelet agents under strict antihypertensive therapy. The prognosis was evaluated using mRS at hospital discharge and 12 months.

The Wilcoxon rank-sum test was performed, and a p-value of less than 0.05 was considered statistically significant.

## Results

A total of 2,776 consecutive patients admitted to our stroke center for ischemic stroke between January 2012 and December 2021 were included, and clinical information was collected and examined. The data are presented in N (%). Of the 2,776 cases of cerebral infarction, 75 (2.7%) were confined to the anterior cerebral artery territory. Among these, ACD was diagnosed in 16 cases (21.3%). At the onset, akinetic mutism was present in four patients (25.0%) and headache in only one patient (6.3%). Table 1 shows the frequency of ACD compared to the previous case series, and Table 2 presents the characteristics of the 16 cases.

**Table 1:** Review of case series literature on infarction due to ACD ACA: anterior cerebral artery; ACD: anterior cerebral artery dissection

Reference	N	Infaction of ACA territory	ACD	ACD / Infarction of ACA territory
Sato et al. (2010) [1]	3115	42 (1.3%)	18	42.9%
Nagamine et al. (2014) [7]	2315	34 (1.5%)	11	32.4%
Present study	2776	75 (2.7%)	16	21.3%

**Table 2:** Characteristics of patients with infarction due to anterior cerebral artery dissection AF: atrial fibrillation; CLZ: cilostazol; DL: dyslipidemia; DM: diabetes; F: female; HT: hypertension; L: left; M: male; mRS: modified Rankin Scale; R: right; +: yes; -: no

Case	Age (years)	Sex	Side	Neurological symptoms					Neuroradiological findings					CLZ	mRS				Past history					
				Hemiplegia	Aphasia	Headache	Aggravation of neurological findings		Intramural hematoma	Double lumen	Aggravation of stenosis	Normalization	Diagnosis basis		At admission	At discharge	After 1 year		HT	AF	DM	DL	Drinking	Smoking
1	45	M	R	+	-	-	-		+	-	+	Residual stenosis	Change in findings	-	3	1	0		-	-	-	-	+	-
2	45	M	R	+	-	-	-		+	-	+	Residual stenosis	Change in findings	-	1	0	0		+	-	-	-	+	+
3	49	F	R	+	-	+	-		+	-	+	After 1 year	Change in findings	-	5	0	0		+	-	-	-	+	+
4	53	F	L	+	+	-	-		+	-	-	After 6 months	Change in findings	+	4	3	2		+	-	-	-	+	+
5	76	M	L	+	+	-	+		-	-	-	Residual stenosis	Change in findings	+	4	5	5		+	-	-	-	+	+
6	59	F	L	+	-	-	-		-	-	+	After 1 year	Change in findings	-	4	4	2		+	-	-	-	-	+
7	42	M	R	+	-	-	-		+	+	+	After 6 months	Double lumen	-	2	0	0		+	-	-	-	-	+
8	60	F	R	+	-	-	-		+	-	-	After 6 months	Change in findings	-	4	1	1		+	-	-	+	-	-
9	54	M	L	+	+	-	-		+	+	+	After 6 months	Change in findings	-	5	4	2		-	-	-	-	+	+
10	76	F	L	+	-	-	-		-	+	+	Residual stenosis	Double lumen	-	4	3	2		-	-	-	-	+	-
11	71	M	R	+	-	-	-		-	-	-	After 1 month	Change in findings	-	4	4	4		+	-	-	-	+	-
12	67	M	R	+	-	-	+		+	-	-	Residual stenosis	Change in findings	+	1	4	2		-	-	-	-	-	-
13	67	M	L	+	-	-	-		-	-	+	Residual stenosis	Change in findings	-	1	1	1		-	-	+	-	-	-
14	75	F	R	+	-	-	+		-	+	+	After 1 year	Double lumen	-	1	4	3		+	-	-	-	-	-
15	56	M	L	+	+	-	-		+	+	+	After 3 months	Double lumen	+	4	4	4		+	-	-	-	+	+
16	51	M	L	+	-	-	-		-	-	+	After 6 months	Change in findings	+	3	0	0		+	-	-	-	+	-

**Figure 1:**
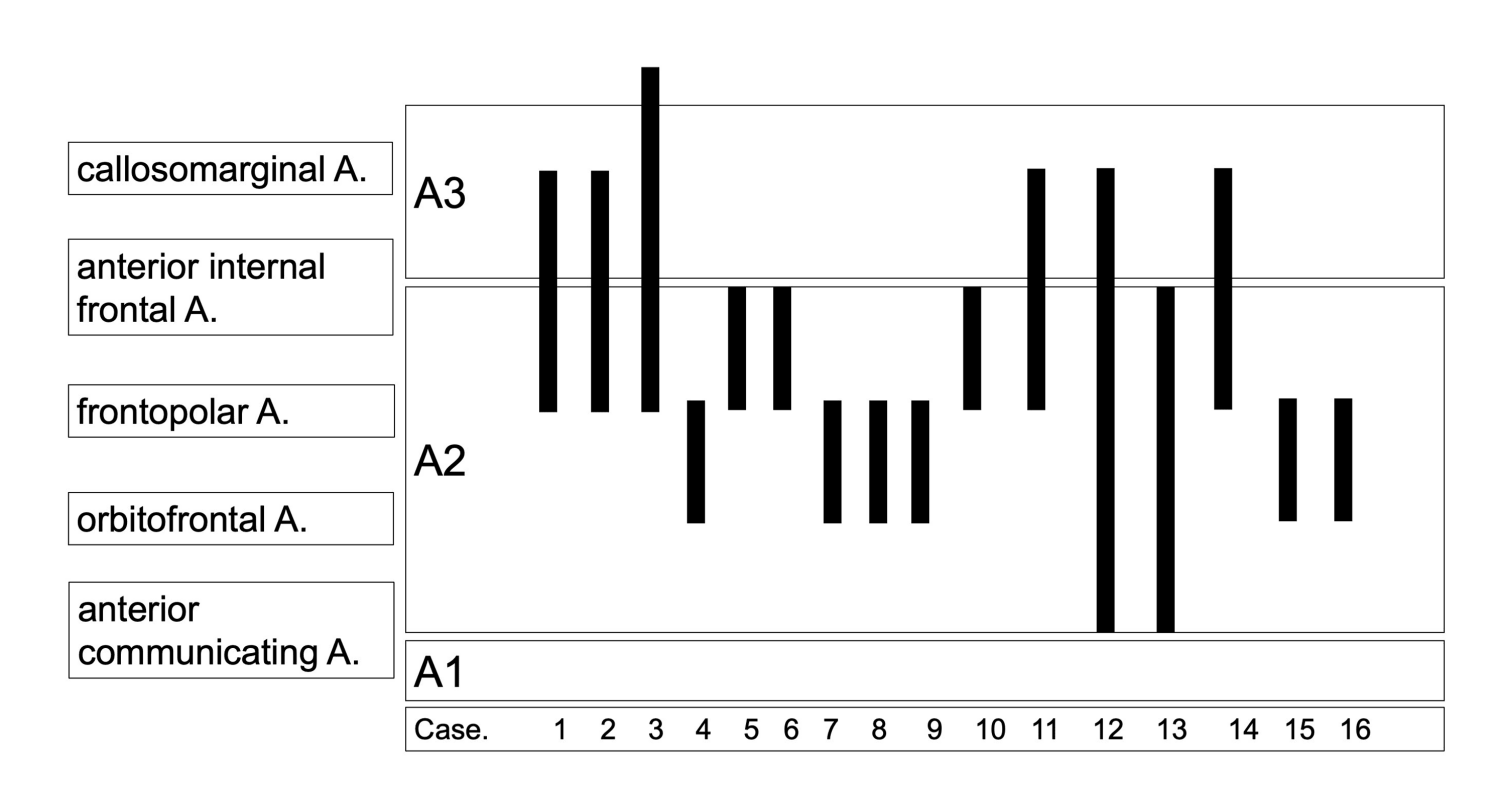
Localization of lesions The horizontal axis indicates each case, and the vertical axis indicates the length of the lesion. We found lesions between the orbitofrontal and anterior internal frontal arteries in all cases. A1 segment: Extends from the internal carotid artery (ICA) to the anterior communicating artery. A2 segment: Extends from the anterior communicating artery (AComA) to the region of the rostrum and genu of the corpus callosum. A3 segment: Runs along the superior aspect of the corpus callosum and extends toward the parietal lobe.

The neurological symptoms were hemiplegia in all patients (100%), akinetic mutism in four (25.0%), dysarthria in nine (56.3%), and the mean National Institutes of Health Stroke Scale (NIHSS) score was 4.88 (two to 12). Headache at onset was present in only one patient (6.3%). Eight patients (50.0%) had left lesions, 15 (93.8%) had enlarged cerebral infarcts on the day after admission, and four (25.0%) had progressive neurological symptoms. Seven (43.8%) patients had an mRS of 0 to two at discharge and 12 (75.0%) at one year. Neuroradiological findings were diagnostic on the first examination in four cases (25.0%), and IMH was detected in 10 cases (62.5%), all at the two-week follow-up. All patients (100%) had changes in findings that supported the diagnosis of ACD. Double lumen was found in four patients (25.0%), and pearl and string in string patients (43.8%). Worsening of stenosis was observed in 11 patients (68.8%), and 11 patients (68.8%) had normalized vascular findings. Figure 1 summarizes the relationship between lesions and branching vessels. The lesions were located beyond A2 (extends from the anterior communicating artery (AComA) to the region of the rostrum and genu of the corpus callosum) in all cases (100%), and the predominant site of involvement was from the post-bifurcation of the orbitofrontal artery to the pre-bifurcation of the anterior internal frontal artery.

A comparison of patients aged ≤60 years and those aged ≥61 years is summarized in Table 3. There were no significant differences in gender, side of the lesion, neurological symptoms, or prognosis, and IMH was not significantly detected in patients aged ≥61 years.

**Table 3:** Bivariate analysis of patients under 60 years of age and other patients

Category	Variable	≤ 60 (10 cases)	> 60 (6 cases)	p-value
Demographics	Female	4	2	0.81
	Left side	5	3	1
Neurological symptoms	Hemiparesis	10	6	-
	Aphasia	3	1	0.57
	Headache	1	0	0.34
	Progression	0	3	0.08
Neuroradiographical findings	Intramural hematoma	8	1	0.01
	Double lumen	3	2	0.9
Modified Rankin Scale (mRS) 0-2	At admission	2	3	0.28
	At discharge	6	1	0.09
	After 1 year	9	3	0.15

Case presentation

Case 8: A 60-Year-Old Female Patient 

**Figure 2:**
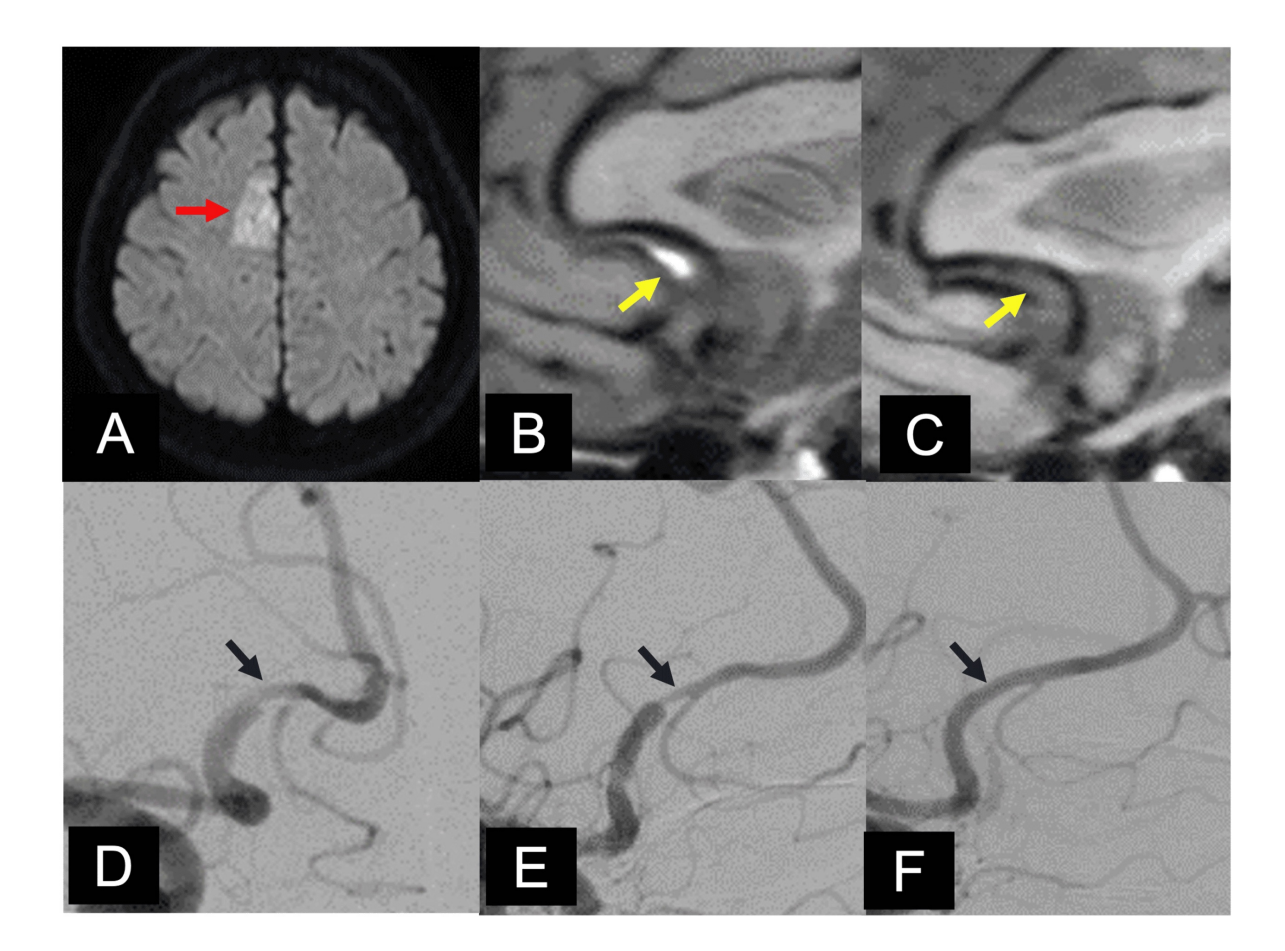
Case 8's radiological images A: The MRI showed acute cerebral infarction in the right anterior cerebral artery (ACA) territory (red arrow). B: We detected intramural hematoma consistent with the lesion using motion-sensitized driven-equilibrium sequence (MSDE). C: MSDE after three months showed the disappearance of intramural hematoma (yellow arrows). D: Digital subtraction angiography (DSA) at admission revealed irregular dilatation and stenosis in the right A2. E: DSA after two weeks showed changes in findings from distal to the bifurcation of the orbitofrontal artery to proximal to the bifurcation of the frontopolar artery. F: DSA after three months showed normalization of ACD findings (blue arrows). A2: Extends from the anterior communicating artery (AComA) to the region of the rostrum and genu of the corpus callosum

The patient presented to our emergency department with paralysis of the left lower extremity, and an MRI showed acute cerebral infarction in the right anterior cerebral artery territory (Figure 2A). The patient was diagnosed with iACD, and conservative treatment was continued. Three months later, the findings of ACD were normalized, and IMH disappeared (Figures 2B, 2E). At the three-month follow-up, the ACD findings normalized and the IMH disappeared (Figures 2C, 2F). Complete paralysis of the left lower extremity has completely recovered, and the patient is following up with mRS 0.

Case 1: A 45-Year-Old Male Patient

**Figure 3:**
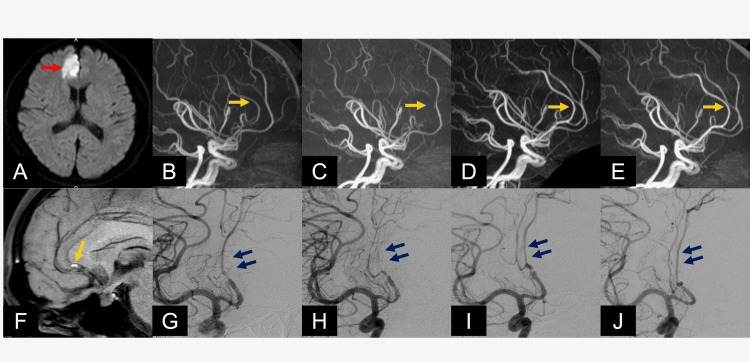
Case 1's radiological images A: An MRI at admission revealed acute cerebral infarction in the right anterior cerebral artery (ACA) territory (red arrow). The magnetic resonance angiography (MRA) showed at admission (B), after two weeks (C), after three months (D), and after one year (E) changes in findings of stenosis of ACD over time (orange arrow). F: Motion-sensitized driven-equilibrium sequence (MSDE) showed high intensity at stenosis of ACA, which suspected intramural hematoma (IMH) (yellow arrow). Digital subtraction angiography (DSA) at admission (G), after two weeks (H), after three months (I), and after one year (J) revealed finally normalization of ACD (blue arrows).

He presented to our emergency department with left hemiplegia with predominant paralysis of the lower extremities, and an MRI revealed an acute cerebral infarction in the right anterior cerebral artery territory (Figure 3A). Based on his DSA findings showing the pearl and string sign on the right anterior cerebral artery, we diagnosed the patient with iACD (Figures 3B, 3G). We continued conservative treatment. Two weeks later, the iACD findings aggravated, with DSA and MRA showing the right pericallosal artery occluded (Figures 3G, 3H). However, surgical treatment was not selected as there was no neurological progression. At this point, MSDE detected IMH at the stenosis area of the anterior cerebral artery (Figure 3F). After three months, ACD findings dramatically improved (Figures 3D, 3I). After one year, the vessel diameter of the anterior cerebral artery normalized (Figures 3E, 3J). The paralysis of the left lower extremity improved with the passage of time, and the patient is currently undergoing outpatient follow-up at mRS 0.

## Discussion

In our series, 2.7% of all cerebral infarctions were confined to the ACA territory, and 21.3% of them were caused by ACD. Compared with previous reports in this series (Table 1) [1, 6], iACD is not a rare condition. In our series, only 25% of the patients were diagnosed at the initial examination, and the rest were diagnosed on the basis of changes in findings or the appearance of IMH at the two-week follow-up. In previous reports, double lumen and intimal flap were diagnosed in 39% to 50% of cases [1, 6-8], and it was considered difficult to identify the anterior cerebral artery because of its small caliber and frequent bending [2]. In a meta-analysis, 89 of 91 cases were reported by Asian patients [2], and Minematsu et al. reported that the second most common intracranial artery dissection after the vertebral artery was the internal carotid artery in Europe and the United States, but ACD was reported in Japan [9].

The mean age of onset was 59.1 years, which is five to eight years older than previous reports, with the highest age being 76 years [6, 9]. The characteristics of patients aged 60 years or older were examined in Table 3, and IMH is difficult to detect, and the older the patient, the more difficult it may be to diagnose.

The common symptom of intracranial artery dissection is headache due to injury to the vessel wall. In previous reports, headache and posterior neck pain were present at onset in 53% to 75% of cases [2, 4], but in our series, only one case (6%) was found. We believe that the diagnosis of ACD could have been made by examining the patient without a headache at the onset of the disease. Half of the patients with left lesions had aphasia, some of which were transient due to supplementary motor area syndrome. Two patients had permanent aphasia, and their mRS was four to five at one year after unsuccessful rehabilitation. Patients with left lesions or aphasia at onset may have a poor outcome, and the course of treatment should be carefully monitored. 

Double lumen was detected in five patients (31%), but as reported in the meta-analysis (32%), double lumen could be detected in less than half of the patients. In the series by Nagamine et al., IMH was detected in 27% of cases [6]. In the present study, IMH was detected in nine cases (56.2%), more than in the previous report (20%) [2]. All of the representative cases disappeared with follow-up over time. Repeated cerebral angiography and MRI are burdensome for patients, but it has been reported that sagittal evaluation by magnetic resonance cisternography is easier to perform [10].

Regarding treatment, since cerebral artery dissection is mainly caused by bleeding within the vessel wall, as indicated by the presence of IMH, intensive hypotensive treatment was administered. Therefore, even if patients are transported within 4.5 hours of onset, we basically do not administer intravenous tissue plasminogen activator (tPA) therapy for suspected iACD, and past reports have stated that its use should be discouraged [11]. We considered the use of antiplatelet agents in patients with multiple risk factors for atherosclerosis. Eleven patients (68.8%) showed progression of stenosis with changes in findings over time, but conservative treatment was continued, and the prognosis was good with mRS 0 to two of 75% at one year. Ten of the patients had normalized findings, and the remaining six had only partial residual stenosis. Tanikawa et al. reported that endovascular or surgical treatment was performed in 10% of cases and was reported to be useful in preventing the progression of ischemic symptoms and the development of bleeding [12]. In our study, patients were treated with antihypertensive therapy and antithrombotic therapy as appropriate, and no transient ischemic attack (TIA) or aneurysm occurred. Hence, we consider antihypertensive therapy to be a very useful treatment for arterial dissection.

We have reached the following conclusions: the rarity of headaches at onset and the presence of ACD in older patients by its longitudinal imaging follow-up, comparison with past literature, and a patient cohort contribute to evolving clinical knowledge. However, limitations include a small sample size from a single institution, which reduces generalizability. The study also relies primarily on imaging rather than histopathology or advanced biomarkers to confirm the ACD diagnosis. Further study and discussion on potential selection bias and how ACD is differentiated from embolic stroke would be needed.

## Conclusions

The possibility of arterial dissection should be taken into consideration when cerebral infarction in the anterior cerebral artery territory is observed. In addition, longitudinal imaging follow-up is useful for diagnosis. Antihypertensive therapy is a useful treatment for anterior communicating artery dissection. Our study differs from previous reports in that the disease also occurs in elderly patients and headache complaints are rare.
